# Fabrication of heterojunctioned Co_3_O_4_-Bi_2_O_3_ nanocomposites and their sustainable electrocatalytic degradation of rhodamine B, and direct red 31 dyes

**DOI:** 10.1039/d6ra04249j

**Published:** 2026-07-29

**Authors:** Elbadawy A. Kamoun, Nourhan A. M. Ragab, Heba Y. Zahran, Faheem Shah, M. Y. Nassar, Esam Bakir, V. Ganesh, Ibrahim S. Yahia

**Affiliations:** a Department of Chemistry, College of Science, King Faisal University Al-Ahsa 31982 Saudi Arabia ekamoun@kfu.edu.sa badawykamoun@yahoo.com; b Nanotechnology and Catalysis Sections, Egyptian Company for Carbon Materials El-Sheraton/El-Nozha Cairo 11757 Egypt; c Laboratory of Nano-Smart Materials for Science and Technology (LNSMST), Department of Physics, Faculty of Science, King Khalid University P.O. Box 9004 Abha Saudi Arabia dr_isyahia@yahoo.com

## Abstract

Heterojunctioned Co_3_O_4_-Bi_2_O_3_ nanocomposite-based electrocatalysts were prepared by the sol–gel/auto-combustion method. The electrocatalytic performance of the NCs towards rhodamine B (RhB) and direct red 31 (DR 31) dyes in water under current–voltage irradiation was evaluated. The prepared Co_3_O_4_-Bi_2_O_3_ NCs were examined by XRD, FT-IR, FE-SEM, EDX, and UV-visible spectroscopy analyses. The formation of the Co_3_O_4_-Bi_2_O_3_ NCs was confirmed by XRD analysis, with crystallite sizes ranging from 52.10 to 57.24 nm. Meanwhile, the FTIR results further proved the formation of a heterostructure, in line with the XRD patterns. FE-SEM investigation presented a spherical Co_3_O_4_ NP aggregate flake structure on the Bi_2_O_3_ NPs. Meanwhile, UV-Vis DRS spectroscopy revealed improved visible-light absorption, leading to a remarkable decline in band gap of ∼2.85–1.87 eV. Notably, the electrocatalytic results showed that the Co_3_O_4_-Bi_2_O_3_ NC coded as CBO-6 could effectively degrade 99.61% of RhB within 120 s and 93.32% of DR 31 within 56 min under electrocatalytic irradiation. Scavenger investigation showed that the creation of ROS, particularly hydroxyl radicals, plays a major role in degrading RhB dye. In the stability test, after the 5th cycle, the RhB dye's degradation efficacy was 94.5%. These findings demonstrate that the CBO NCs are appropriate nanomaterials for RhB electrocatalytic degradation, including dye breakdown into carbon dioxide and water.

## Abstract 2

Heterojunctioned Co_3_O_4_-Bi_2_O_3_ nanocomposite-based electrocatalysts were prepared by the sol–gel/auto-combustion method.

## Graphical abstract

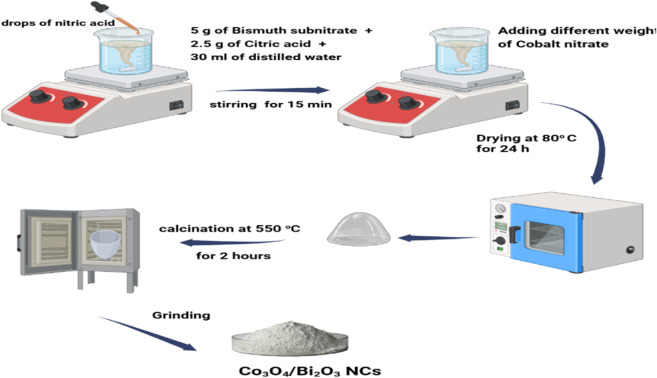

## 1 Introduction

Pollution has become a serious issue that endangers the lives of people and other living things, owing to recent industrial development. Over 2.3 billion people suffer from water-related illnesses, making water pollution one of the most critical environmental contamination issues.^1^ Because of their destructive impact on the environment, wastewater containing organic contaminants, such as textile dyes, is a source of concern.^2^ One of the more significant categories of contaminants found in wastewater discharged by the textile industry and other sectors is organic dyes. Environmental issues include aesthetic pollution (even a small amount of dye is readily visible) and the disturbance of aquatic life that arises when highly colored wastewater is released into the ecosystem.^3^ Direct red 31 (DR-31) and rhodamine B (RhB) are two examples of organic dyes. Because of its toxicity and resistance to natural degradation, rhodamine B (RhB), a persistent organic pollutant, poses serious ecological risks.^4^ RhB is frequently used as a pigment in culinary products, textiles, paper, and printing. Because of its toxicity profile, releasing it into the environment as wastewater poses major health risks. It causes neurotoxicity and carcinogenicity. Symptoms of RhB exposure include nausea, vomiting, respiratory issues, and gastritis, and it is difficult to degrade.^5^ When dissolved in water, the organic red-brown powder known as direct red 31 (DR-31) turns crimson, named bis-(4-hyroxy-2 sulfonathalen-7-yl) amine. It is used to color a variety of textiles, including silk, wool, paper, and leather, and it is insoluble in organic solvents.^6^ Despite this, DR-31 dye has negative consequences for human health. Because of its soluble nature, several researchers are using various approaches.

As a result, scientists are concentrating on finding solutions to environmental contamination and eliminating harmful water pollutants. As a result, they are working on developing cutting-edge methods for treating wastewater. One effective technique for treating wastewater is dye degradation. Filtration, flocculation, adsorption, chlorination, and stripping are all steps in the dye degradation process.^7–11^ Alternative techniques for purifying these fluids are under extensive research. Water purification can benefit from the mineralization of organic molecules using advanced oxidative processes (AOP),^12–14^ which raised through environmental elements and require expensive regeneration.^11^ Complex equipment is required for advanced oxidation processes (AOPs), which operate under extreme working conditions.^12^ On the other hand, because of its superior controllability, mild reaction conditions, and the lack of secondary pollutants during electrolysis, electrocatalytic oxidation has attracted increased attention.^13^ The production of hydroxyl radicals, electrocatalytic activity, and service life are all directly impacted by the anode materials, which are crucial components in the electrocatalytic breakdown of resistant organic molecules.^15,16^ One of the dependable techniques for dye degradation is electrocatalysis. With a simple setup consisting of a cathode and anode plate plunged in electrolyte and coupled to a DC electrical power source, electrocatalysis is widely used to break down contaminants in wastewater. When charged ions in circuits acquire or lose electrons, compounds degrade. This approach has several advantages over competitors, including being more environmentally friendly, using less energy, being flexible, being appropriate for situations with significant pollution, and being simple to use.^17^ Recently, bismuth oxide (Bi_2_O_3_) has attracted significant interest, owing to its high electrical conductivity and small bandgap (2.8 eV).^18^ It is an intriguing material for the electrochemical oxidation of several organic contaminants, as it offers a wide surface area, electrochemical stability, and catalytic activity.^19^ The fast combination of electron–hole (e^−^/h^+^) pairs produced throughout the electrocatalytic reaction is the main cause of the ongoing difficulty with the practical deployment of single Bi_2_O_3_. Numerous tactics have been put forth and employed to get around these boundaries. Among these, doping with semiconductors to create heterojunctions, such as Bi_2_O_3_-BiOBr and Co_3_O_4_-Bi_2_O_3_, has been widely used as a potential strategy.^20^ Due to its various properties, cobalt (ii, iii) oxide spinel (Co_3_O_4_) is appealing and has attracted extensive research interest in recent years. The high activity and selectivity of metal oxide catalysts are believed to be due to variations in oxygen hole density, oxygen defects, and oxygen adsorbed in various cobalt states.^21,22^ Additionally, cobalt (ii, iii) oxide is a p-type semiconductor that offers acceptable catalytic degradation possible for organic pollutant wastewater, due to its intriguing electronic, optical and magnetic characteristics, low solubility, high thermal/chemical stability, and slight bandgap within 1.2–2.1 eV.^21,23^ Therefore, Co_3_O_4_ can be considered as an ideal modifier to enhance the performance of Bi_2_O_3_.^24^ Because of its narrow band gap, adding Co_3_O_4_ to Bi_2_O_3_ could greatly increase the electrocatalytic activity for the degradation of organic contaminants by enhancing the charge separation at the interface.^25^

This work describes the fabrication of Co_3_O_4_-Bi_2_O_3_ NCs as efficient electrocatalysts for the degradation of rhodamine B and direct red dyes. A sol–gel/auto-combustion technique was successfully used for the preparation of pure Bi_2_O_3_ NPs and Co_3_O_4_-Bi_2_O_3_ NCs. XRD, FT-IR, FE-SEM, elemental mapping, and UV-DRS methods were utilized to check the physical and chemical characteristics of the produced nanocomposites. This novelty is based on the creation of a p–n heterojunction between Co_3_O_4_ and Bi_2_O_3_, which significantly enhances the charge separation and electron transport efficiency. The synergistic interaction between redox-active Co^2+^/Co^3+^ centers and Bi_2_O_3_ leads to superior degradation performance by promoting the generation of reactive oxygen species. The electrocatalytic degradation activity of the produced nanocomposites was investigated using direct red 31 (DR 31) and rhodamine B (RhB) as models for wastewater treatment functions.

## 2 Materials and methods

### 2.1 Materials

Bismuth subnitrate, Bi_5_H_9_N_4_O_22_; cobalt nitrate, Co(NO_3_)_3_·9H_2_O; citric acid, C_6_H_8_O_7_; nitric acid, HNO_3_; distilled water; direct red 31 dye, C_32_H_21_N_5_Na_2_O_8_S_2_; rhodamine B, C_28_H_31_ClN_2_O_3_; ascorbic acid, C_6_H_8_O_6_; isopropyl alcohol, C_3_H_8_O; sodium chloride; ethylenediamine tetraacetic acid (EDTA), C_10_H_16_N_2_O_8_; and sodium nitrate, NaNO_3_, were used.

### 2.2. Synthesis of the Co_3_O_4_-Bi_2_O_3_ nanocomposite

**Fig. 1:**
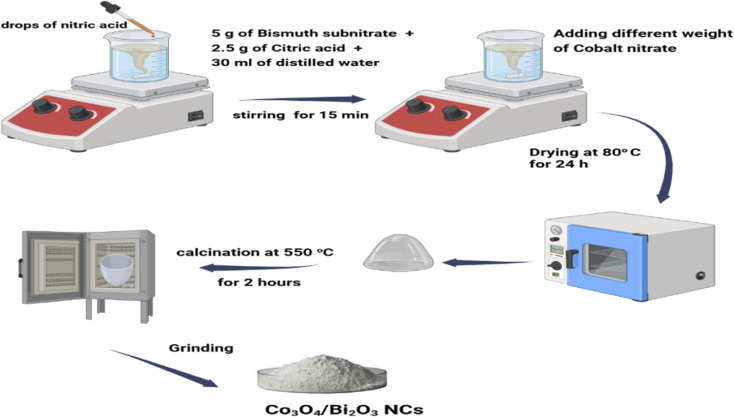
Schematic diagram of preparation of Co_3_O_4_/Bi_2_O_3_ NCs.

The Co_3_O_4_-Bi_2_O_3_ nanocomposite was synthesized *via* the sol–gel/auto-combustion method *via* bismuth subnitrate, and citric acid was employed as an oxidant and fuel. A fixed mass of 5 g of bismuth subnitrate, 2.5 g of citric acid, and a few drops of nitric acid, were each dissolved in a crucible to obtain a homogeneous solution. Cobalt nitrate was used as the Co_3_O_4_ dopant source, introduced in varying concentrations (0, 0.001, 0.01, 0.1, 0.25, 0.5, and 1.0 wt%) relative to Bi_2_O_3_. Each dopant concentration was dissolved in 30 mL of DW and mixed with the precursor solution under constant stirring. The mixture was heated to 80 °C and kept at 80 °C for 24 h in a drying oven to initiate gelation. The resultant gel was then heated to 550 °C in a muffle furnace for 2 h. The resultant NC was allowed to cool, followed by gentle grinding in an agate mortar and pestle to produce a fine powder, as shown in Fig. 1. The resultant Co_3_O_4_-Bi_2_O_3_ NC powders were coded as shown in Table 1.

**Table 1:** Sample codes of the prepared Co_3_O_4_-Bi_2_O_3_ NCs

Sample	Code
Pure Bi_2_O_3_	CBO-0
0.001 Co_3_O_4_-Bi_2_O_3_	CBO-1
0.01 Co_3_O_4_-Bi_2_O_3_	CBO-2
0.1 Co_3_O_4_-Bi_2_O_3_	CBO-3
0.25 Co_3_O_4_-Bi_2_O_3_	CBO-4
0.5 Co_3_O_4_-Bi_2_O_3_	CBO-5
1.0 Co_3_O_4_-Bi_2_O_3_	CBO-6

### 2.3 Electrocatalytic measurement

The catalytic activities of the CBO-0 NPs and CBO NCs under electrocatalytic conditions were investigated using the dyes rhodamine B (RhB) and direct red 31 (DR31). The starting concentrations of the RhB and DR31 dyes were 0.01 mg L^−1^ and 0.03 mg L^−1^, respectively. The electrocatalytic activity of two graphite electrodes was measured to assess their ability to degrade dyes. A 5 volt DC power supply was used to set up two-electrode electrochemical cells. The cell was filled with 200 mL of water solution, plus RhB and DR31, respectively, with the cathode and anode positioned 2 cm apart. The conductivity increased upon adding 10 milliliters of 1.0 M NaCl. The dye solution in a single-cell reactor was fully mixed before 0.01 g of CBO NCs were added. To find the adsorption–desorption equilibrium, the solution was left in the dark for 30 min, as presented in Fig. S1 (SI). The degree of dye degradation was tracked using a UV-visible spectrophotometer. To investigate the electrocatalytic degradation of the dyes, the catalyst makes it easier for charge carriers to move along the external circuit.^26^1
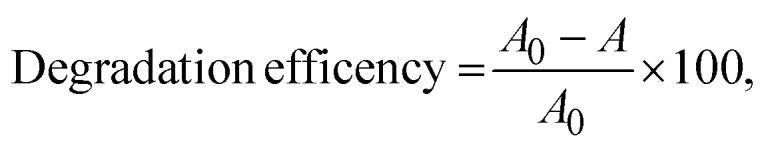
where *A*_0_ is initial absorbance of the dyes, and *A* is their absorbance at specific times.

### 2.4. Characterization and devices

At a scanning rate of 0.02° per second, the CBO NC reflection-mode XRD patterns were recorded (Shimadzu LabX-XRD-6000, Japan) with CuKα emission. In the 2*θ* range of 10–80°, the XRD tube was worked at 30 mA and 30 kV. X'pert High Score software was utilized to investigate and index the XRD peaks. Morphological alternation of the CBO NCs was investigated by FE-SEM apparatus (Vega 3 SBU, Tescan, Czech Republic) with a gold (Au) coating and at a voltage of 30 kV. Elemental mapping units, often known as EDX gadgets, were utilized. ImageJ software was used to determine samples' grain sizes.

The chemical structure of the CBO NCs was estimated by FT-IR spectrometer (Thermo Fisher Scientific, Nicolet 6700, Waltham, MA, USA) covering a spectral range of 4000–400 cm^−1^ with a spectral resolution of 6 cm^−1^ in transmittance mode. Diffuse reflectance spectra (DRS) of the NCs under study were measured by a spectrophotometer (Shimadzu UV-vis-NIR 3600, Japan) at *λ* 300–900 nm, where BaSO_4_ was used as a reference. A JASCO UV-Vis single-beam spectrophotometer was applied to follow the electrocatalytic degradation process at RT. All synthesized samples were entirely prepared and characterized at the Egyptian Company for Carbon Materials (ECCM) in Cairo.

## 3 Results and discussion

### 3.1 XRD analysis

**Fig. 2:**
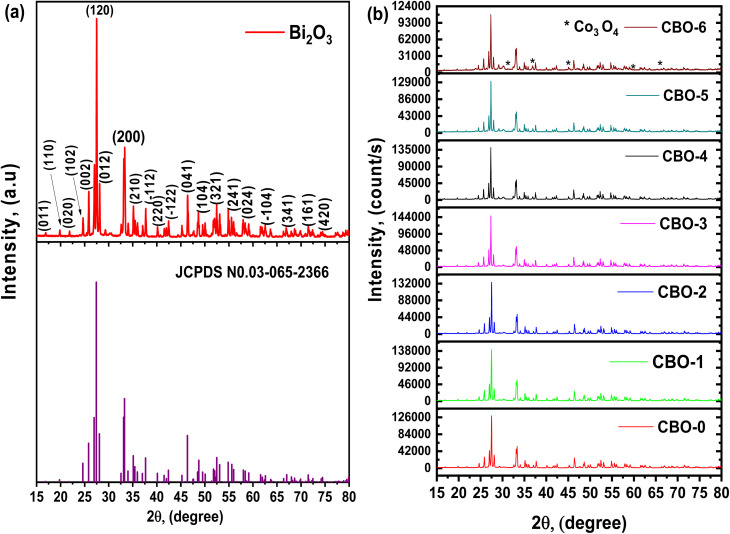
XRD patterns of the (a) Bi_2_O_3_ NPs, and (b) Co_3_O_4_-Bi_2_O_3_ NCs with different concentrations of Co_3_O_4_.

XRD examination of the pure Bi_2_O_3_ NPs and Co_3_O_4_-Bi_2_O_3_ nanocomposites was used to determine their crystal structure, as shown in Fig. 2(a and b). The XRD peaks at 21.73°, 25.84°, 27.15°, 28.04°, 33.24°, 35.02°, 46.39°, 52.36°, and 54.98° are specifically allocated to the (020), (002), (120), (012), (200), (210), (041), (321), and (241) planes of Bi_2_O_3_ NPs, as displayed in Fig. 2(a). The diffraction peaks match with card (PDF #No. 03-065-2366), confirming the monoclinic crystal structure of Bi_2_O_3_ with space group (*P*2_1_/*c*) and lattice parameters of *a* = 5.38 Å, *b* = 8.134 Å, *c* = 6.89 Å.^27^ The cubic Co_3_O_4_ nanostructures show sharp peaks at 2*θ* = 18.94°, 31.12°, 36.80°, 44.30°, 59.96°, and 65.08° corresponding to the (111), (220), (311), (400), (511) and (440) planes. The produced pristine Co_3_O_4_ nanostructure's XRD pattern agrees well with (PDF #No. 65-3103).^28^ XRD examination confirms the development of Co_3_O_4_-Bi_2_O_3_ NCs (Fig. 2(b)). The diffraction peak of Co_3_O_4_ is not observed at the low concentration in the CBO-3 NCs, because the Co_3_O_4_ content is below the XRD detection limit and its weak diffraction peaks overlap with the intense reflections of Bi_2_O during the preparation process; while at high concentrations the peaks for the (311) plane shift to lower diffraction angles. In this case, we can confirm that the pattern for Co_3_O_4_ is possibly due to the weak intensity of the diffraction peak of cobalt oxide, compared to that of bismuth-containing oxides.^29^ Meanwhile, at higher concentrations from the CBO-4 to CBO-6 NCs, interactions between the Co_3_O_4_ and Bi_2_O_3_ nanostructures may be responsible for the modest shift in the XRD peaks towards lower diffraction angles and the decline in peak intensity as the Co_3_O_4_ concentration in the nanocomposite increases.^30^ Ding *et al.*,^31^ found that the addition of Co^3+^, which has an insignificant ion radius (0.063 nm) compared to Bi^3+^ (0.117 nm), is the cause of this minor change. The unit cell volume (*V*) of the fabricated samples and the lattice constants (*a*, *b*, *c*, and *β*) of the monoclinic structure with lattice angle (*α* = *γ* = 90°, *β* = 113°) were estimated from the diffraction peaks through eqn (2)–(4):^32^

2 2*d*_*hkl*_ sin *θ* = *nλ*

3
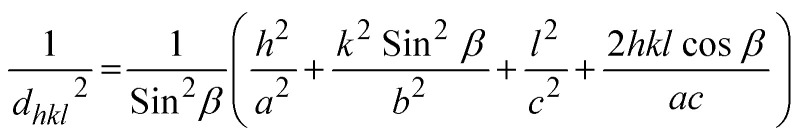

4 *V* = *abc* sin *β*

where *θ* is Bragg's diffraction angle, *λ* is the wavelength of CuKα radiation used, and *d*_*hkl*_ is the interplanar spacing, *h-k-l* are Miller indices, and *a-b-c* are lattice parameters. Scherrer's formula, which was applied to guess the size of the average crystallites, is provided in eqn (5):^33^5
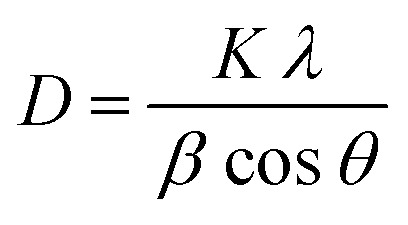
where *θ* is Bragg's angle in radians, *β* is the full width at half maximum (radians), *D* is the crystallite size in nanometers, and *θ* is the wavelength in nanometers. According to Scherrer's method, the typical crystallite size of the synthesized materials ranges from 52.10 to 57.24 nm. Scherrer's method yielded an average crystallite size of 53.05 nm for the CBO-6 NCs and 54.69 nm for the CBO-0 NPs. The variation in crystallite size implies that Co_3_O_4_ influences Bi_2_O_3_ growth. This implies that the effects of lattice strain and crystallite growth are dominant at the oxide interface. One of the popular crucial metrics used to define powder samples is micro-strain (*ε*), which characterizes changes in the particles' size, shape, and microstructure, and is given by eqn (6):^34^6
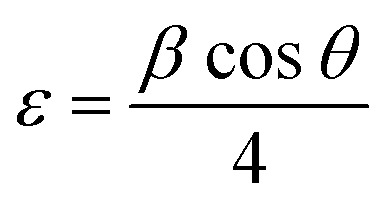


Eqn (7) was used to calculate the dislocation density (*δ*) of the CBO NCs, which indicates the degree of crystallographic defects or randomness inside the crystal structure:^35,36^7
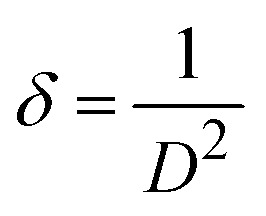


Table 2 displays the computed lattice and structural properties. The good crystallinity of the CBO NCs produced by the sol–gel/auto-combustion process is confirmed by the tiny values of *δ* and *ε* found in this work.

**Table 2:** Crystallite size, dislocation, micro-strain, and crystallographic parameters of the as-synthesized CBO NCs; the tabulated data were calculated from XRD analysis

Sample	CBO-0	CBO-1	CBO-2	CBO-3	CBO-4	CBO-5	CBO-6
*D*, (nm)	54.69	52.10	55.32	57.24	55.27	54.34	53.05
*δ* × 10^−4^, (nm^−2^)	4.59	6.95	4.44	4.80	4.40	5.82	6.58
*ε* × 10^−4^	6.96	7.72	6.88	6.89	6.87	7.43	7.71
*a*, (Å)	5.38	5.379	5.374	5.403	5.390	5.399	5.379
*b*, (Å)	8.134	8.133	8.127	8.194	8.165	8.185	8.135
*c*, (Å)	6.89	6.889	6.888	6.936	6.926	6.928	6.893
*V*, (Å)^3^	277.543	277.417	276.915	282.661	304.808	280.577	277.647

### 3.2. FTIR analysis

**Fig. 3:**
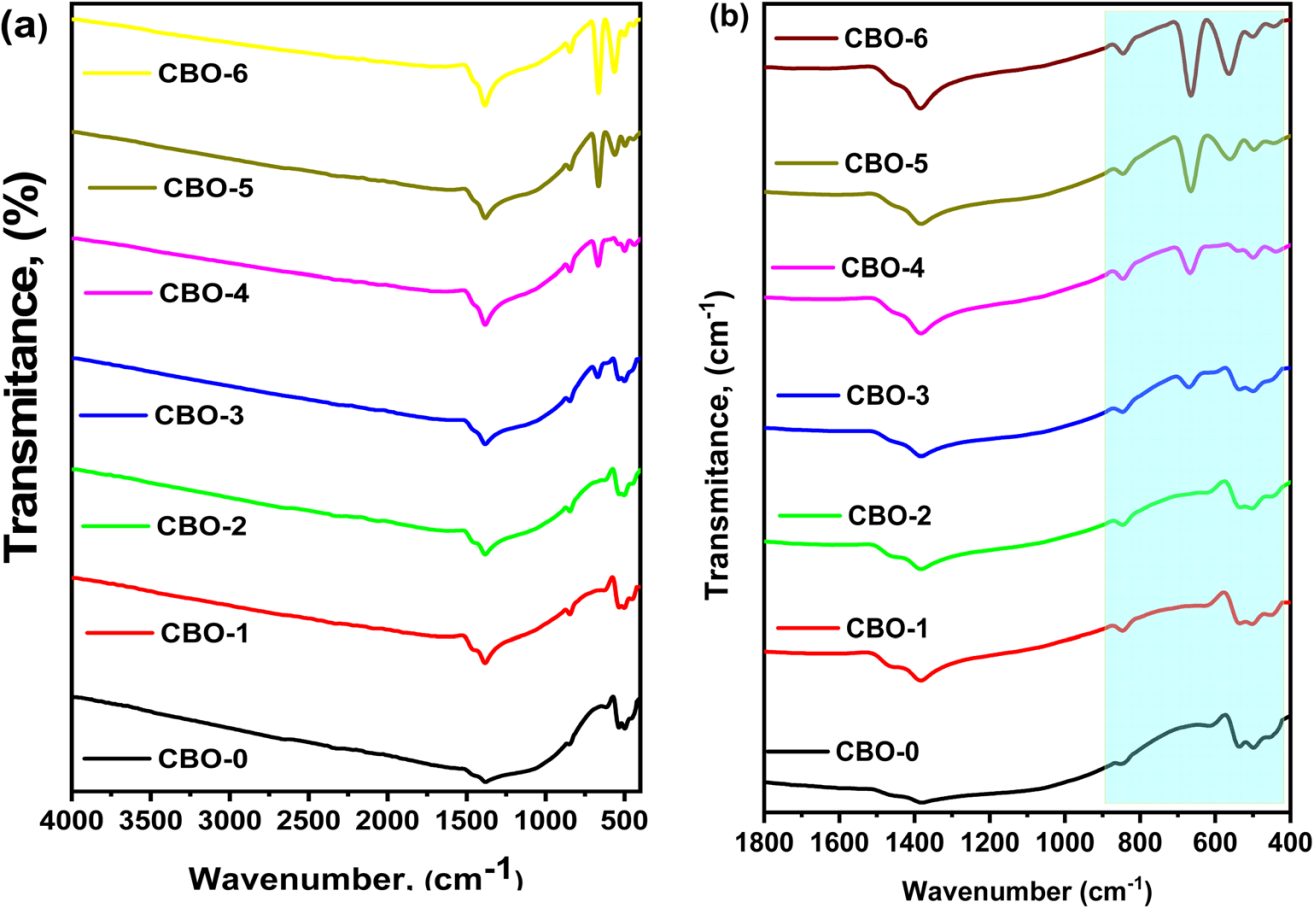
FT-IR spectra of the CBO-0 NPs and CBO NCs with different concentrations of Co_3_O_4_; (a) *ν* 4000–400 cm^−1^ and (b) *ν* 1800–400 cm^−1^.

The chemical bonding and quality of the as-prepared Co_3_O_4_-Bi_2_O_3_ NCs at *υ* 4000–400 cm^−1^ were examined using FT-IR spectroscopy. The raw FTIR-transmittance spectra of the Co_3_O_4_-Bi_2_O_3_ NCs are shown in Fig. 3(a and b), and these featured IR bands are listed in Table S1 (SI). It shows two absorption peaks at *υ* 844 and 564 cm^−1^, which are stretching vibration modes of Bi–O bonds of the BiO_6_ octahedron.^37^ Metal–oxygen vibration modes of Co-O are responsible for the high intensity peaks at *ν* 561 and 665 cm^−1^. The sharp absorption peak at *ν* 1386 cm^−1^ might be due to C–O vibration modes of citric molecules that might be absorbed during the Co_3_O_4_-Bi_2_O_3_ NC preparation process.^38^

### 3.3. FE-SEM investigation

**Fig. 4:**
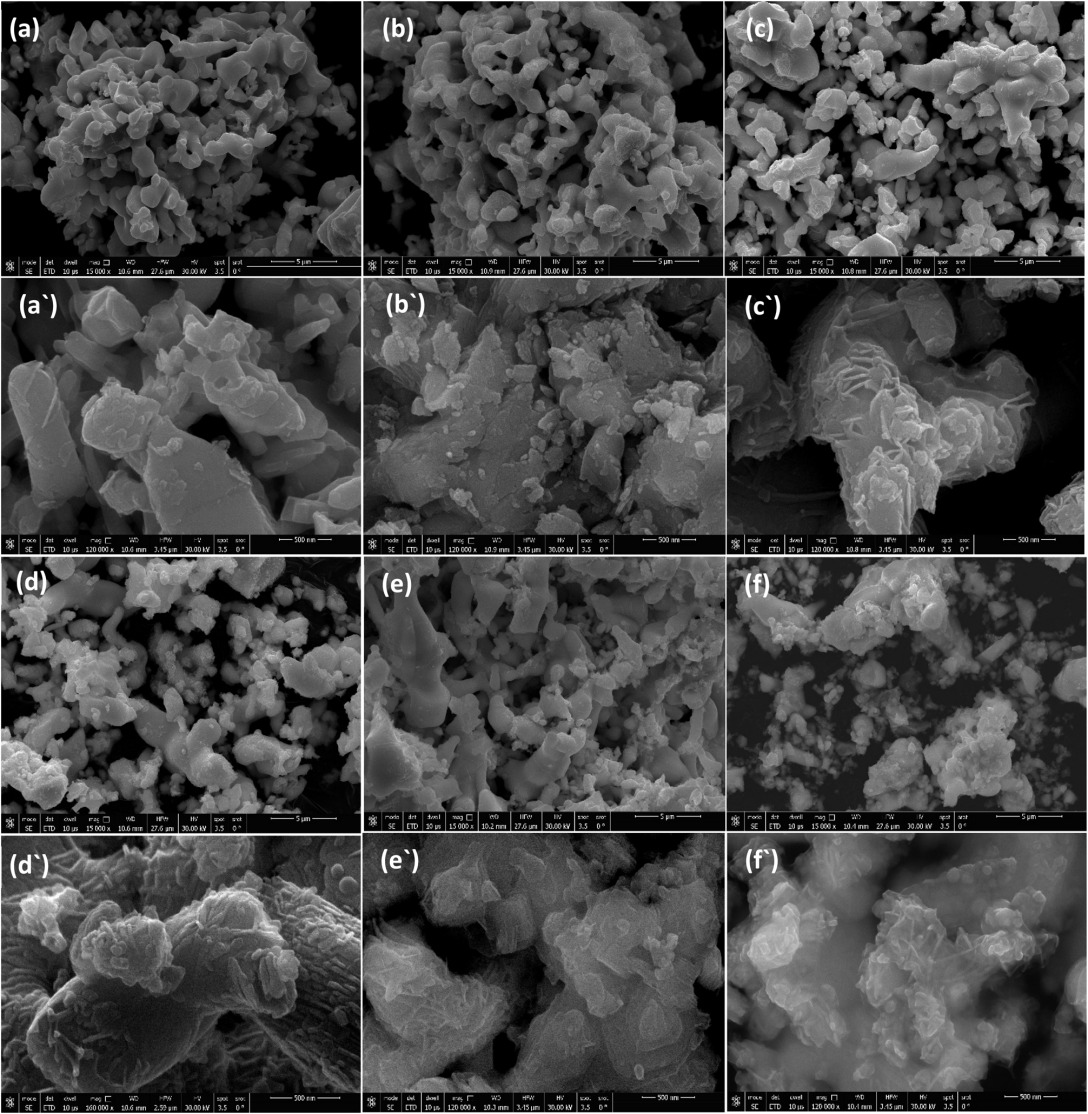
(a–f) SEM micrographs of the CBO-0 NPs and CBO NCs at scale 5 µm, original magnification 150 KX, and applied voltage 30 kV, and (a′–f′) SEM images of the CBO-0 NPs and CBO NCs at scale 500 nm, original magnification 120 KX, and 30 kV.

**Fig. 5:**
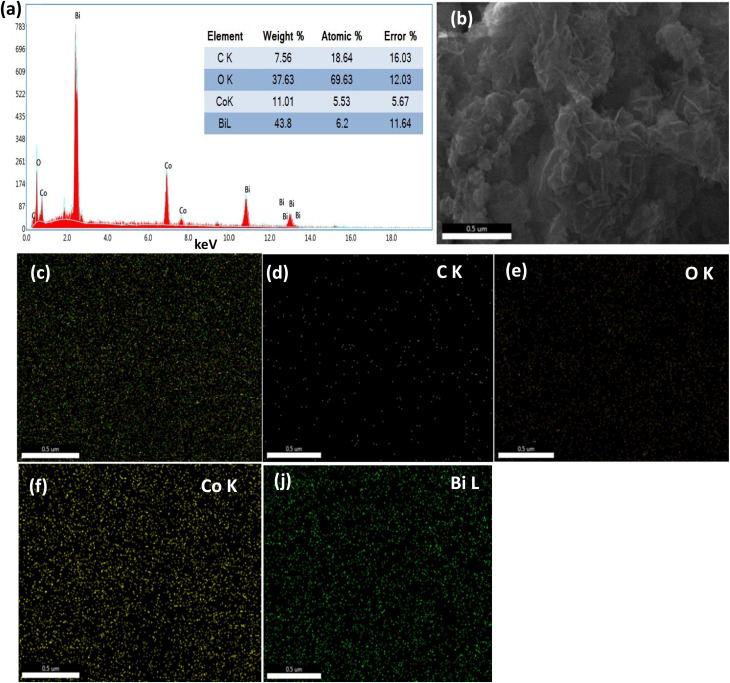
(a) EDX spectrum, and (b–j) elemental mapping analysis of selected CBO-6 NCs.

The morphology of the as-prepared Co_3_O_4_-Bi_2_O_3_ NCs was investigated by FE-SEM investigation of the samples under different magnifications, and the images are shown in Fig. 4(a–f). A detailed examination of the surface morphology of the CBO-0 NPs in Fig. 4(a) reveals that the CBO-0 NPs have the same flake structure morphology as the as-prepared Bi_2_O_3_ NPs, but with a higher density and with random orientation. Fig. 4(a′) presents a random articulated flake structure at 500 nm scale. The diameter of the CBO-0 NPs is found to be in the range between 57 and 165 nm with an average diameter of 92 nm and a length of around 348 nm. Fig. 4(b–f) show the Co_3_O_4_-Bi_2_O_3_ NCs, potentially owing to the growth of Co_3_O_4_ NPs on the Bi_2_O_3_ surface, and strongly evidence that there is interaction between the individual components of the NCs.^39^Fig. 4(b′–f′) present the spherical Co_3_O_4_ NP aggregate flake structure at a scale of approximately 500 nm. EDX with elemental mapping analysis, as shown in Fig. 5(a–j), detected only Co, Bi, and O. The current findings confirm the formation of well-integrated CBO-6 NCs, with distinct morphological features and a homogeneous elemental distribution, in excellent agreement with the XRD results.

### 3.4. DRS analysis

**Fig. 6:**
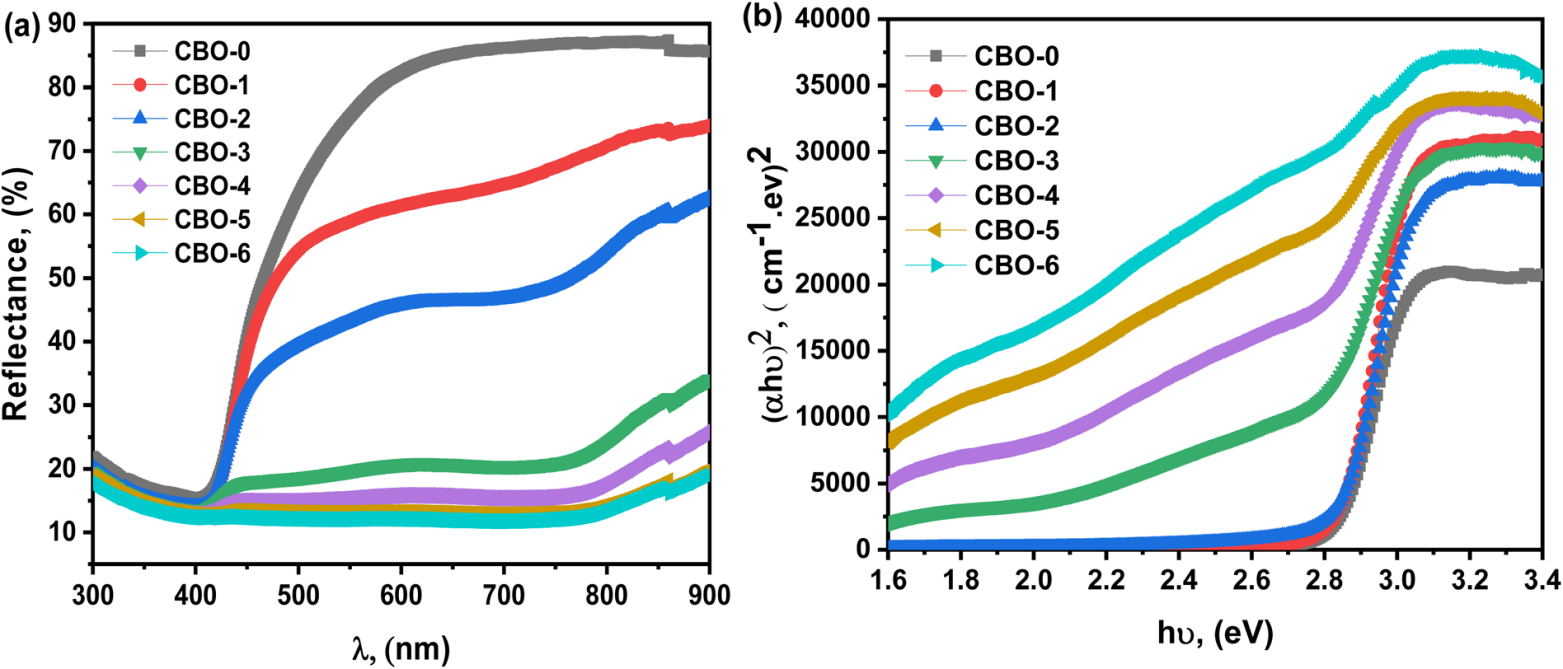
(a) UV-Vis DRS spectra of the CBO NCs, and (b) Tauc plot of the CBO NCs.

Diffuse reflectance UV-Vis spectroscopy was used to assess the optical characteristics and band gap energy of the Co-friendly produced Bi_2_O_3_ NPs, Co_3_O_4_-Bi_2_O_3_ NCs. According to the findings, Bi_2_O_3_ NPs have an absorption edge at *λ* 416 nm, which suggests a large band gap and limited use of visible light.^40^ It is interesting to note that, as shown in Fig. 6(a), the Co_3_O_4_-Bi_2_O_3_ NCs exhibit higher light absorption capacity than the Bi_2_O_3_ NPs toward the visible light area with the absorption edge at 688 nm. On the other hand, the structure and mixed-valence Co^2+^/Co^3+^ electronic transitions of Co_3_O_4_ cause larger absorption throughout the visible spectrum. Increasing the concentration of Co_3_O_4_ nanostructures to Bi_2_O_3_ NPs increases light absorption. The maximum visible-light absorption was observed for CBO-6 NCs. Consequently, the Co_3_O_4_-Bi_2_O_3_ NCs produced material with improved light-harvesting capabilities that extended into the visible range, providing potential benefits for a range of electrocatalytic applications.^41^ The diffuse reflectance process for the powdered samples can be theoretically described by Kubelka–Munk theory. The Kubelka–Munk function *F*(*R*) provides a connection between the absorption coefficient (*α*), and reflectance (*R*):^42^8
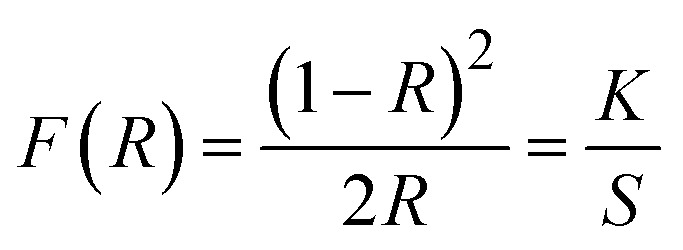
where *R* is reflectance, *K* is molar absorption coefficient, and *S* is scattering coefficient. Tauc's equation sets a relationship between band gap (*E*_g_) and absorption coefficient (*α*), and is given by:^42^

9 (*αhυ*)^*n*^ = *A*(*hυ* − *E*_g_)

where *E*_g_ is the bandgap energy, *α* is the absorption coefficient, *A* is the constant associated with mass of electrons, *n* = 2 for a direct allowed transition, *h* is Planck's constant, and *υ* is frequency. It provides a straight-line plot, and a decent estimate of the band gap is achieved from the intercept of the tangent with the *x*-axis. The bandgap energy values of the Bi_2_O_3_ NPs and Co_3_O_4_-Bi_2_O_3_ NCs were assessed by Tauc plot (Fig. 6(b)). The Bi_2_O_3_ NPs have a varied band gap (2.85 eV), consistent with earlier published values.^43^ In the case of the CBO-6 NCs, the band gap was lower than that of the Bi_2_O_3_ NPs, with an optical band gap of 1.87 eV, as shown in Table 3.

### 3.5. Electrocatalytic activity

**Fig. 7:**
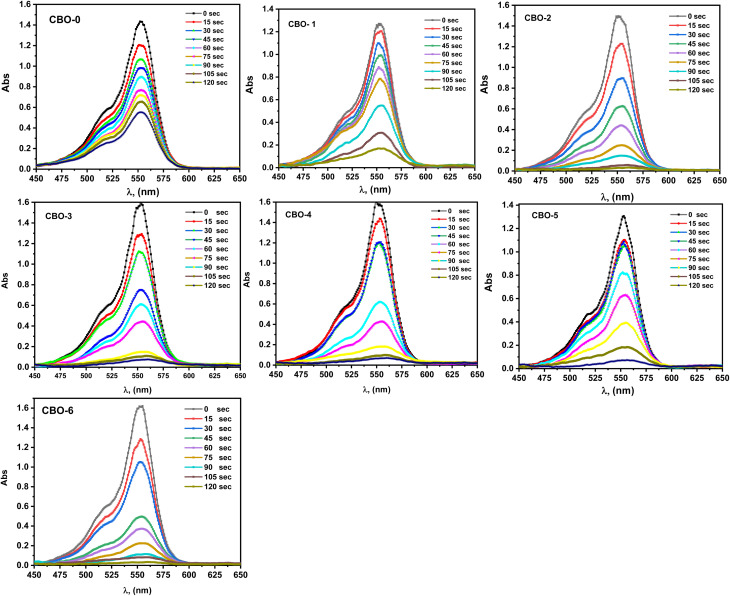
UV-Visible RhB dye spectra with CBO NCs.

**Fig. 8:**
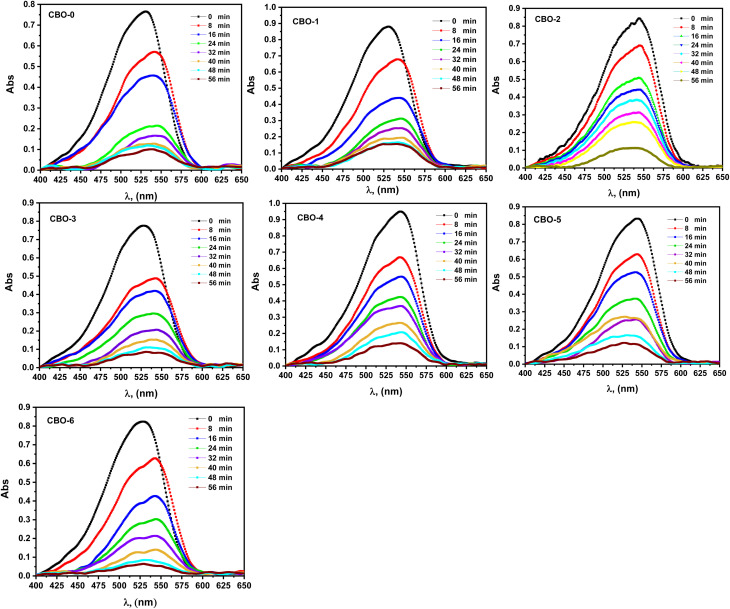
UV-Visible DR-31 dye spectrum with CBO NCs.

**Fig. 9:**
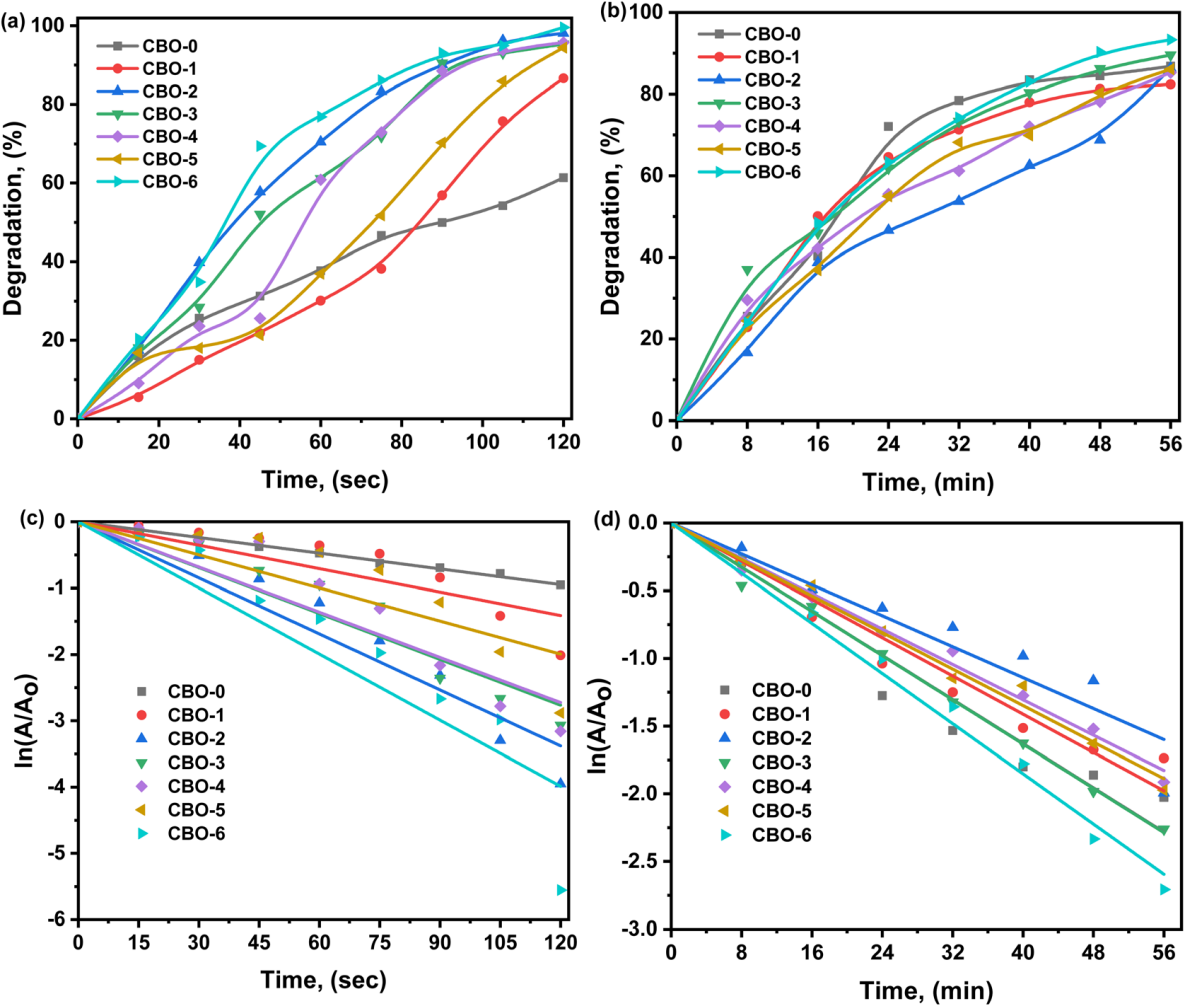
Electrocatalytic efficiency of (a) RhB dye and (b) DR 31 dye, and pseudo-first order kinetic fitting curves of (c) RhB dye, and (d) DR 31 dye with the CBO NCs.

In electrocatalytic degradation reactions, RhB and DR-31 molecules were used as model dye pollutants. Several nanocomposites' chemical properties were examined using UV-visible spectroscopy. CBO-0 NPs and CBO NCs were used in UV-visible spectroscopy to assess the absorption of light for RhB and DR-31 dye degradation at different time intervals. Fig. 7 and 8 show the UV-visible absorption spectra of the RhB and DR-31 dyes with CBO NCs, measured over *λ* 400–650 nm. There are absorbance peaks for RhB and DR-31 at 550 nm and 540 nm, respectively. The findings clearly demonstrate that the electrocatalytic degradation of RhB and DR-31 dyes is greatly increased by the presence of Co_3_O_4_ nanostructures at greater concentrations. The degradation experiment results are consistent with the bandgap results. Electrocatalytic degradation activity rises as the produced nanocomposite's bandgap energy decreases. The nanocomposite's quick electron–hole pair recombination will be facilitated by the low bandgap.^44,45^ The results reveal that all the synthesized electrocatalysts exhibit degradation capabilities. The CBO-0 NPs degraded 61.37% and 86.80% of RhB and DR 31 dye in 120 s and 56 min, respectively. However, in the presence of CBO-6, the NCs degraded 99.61% and 93.32% of RhB and DR 31 dye in 120 s and 56 min, as depicted in Fig. 9(a and b), respectively. Hence, coupling Co_3_O_4_ with Bi_2_O_3_ to form a heterostructure has a crucial effect on the degradation rate of organic dyes, compared to pure Bi_2_O_3_.

### 3.6. Kinetic studies

Kinetic studies of the NCs and RhB and DR 31 dyes were performed using the Langmuir–Hinshelwood model. The experimental data were validated with the pseudo-first-order kinetic model. Due to low initial absorbance of RhB and DR 31 dyes, this model is described as follows:^46^10
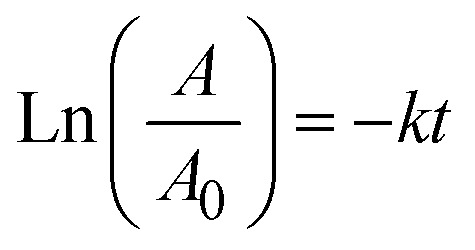
where *A*_0_ is the initial absorbance of the dyes, *A* is the absorbance at any time, *t* is the time in minutes, and the slope of the 
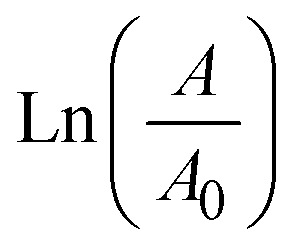
*vs.* time plot gives the value of *k*, the rate constant (min^−1^) of the reaction. The respective dynamic plots of 
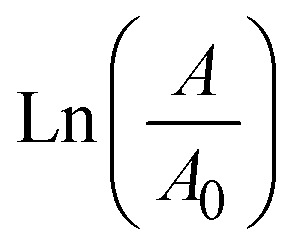
*versus* the irradiation time (*t*) of the RhB and DR 31 dyes are displayed in Fig. 9(c and d), and Table 3 shows the kinetics rate constants of the dyes with different CBO NCs. The CBO-6 NCs had the largest kinetic rate constants, with values of 0.03324 s^−1^ (*R*^2^ = 0.929) and 0.04632 min^−1^ (*R*^2^ = 0.996), respectively, for RhB and DR 31.

**Table 3:** Direct optical band gap, electrocatalytic degradation efficiency, and pseudo-first-order kinetics parameters for RhB and DR 31 dyes under electrocatalytic degradation

Sample	*E*_g_, (eV)	Electrocatalytic degradation of RhB			Electrocatalytic degradation of DR 31		
		Degradation, (%)	*K*, (s^−1^)	*R* ^2^	Degradation, (%)	*K*, (min^−1^)	*R* ^2^
CBO-0	2.85	61.37	0.00789	0.996	86.80	0.04074	0.982
CBO-1	2.83	86.63	0.01177	0.881	82.39	0.03530	0.987
CBO-2	2.81	98.07	0.02817	0.969	86.36	0.02855	0.966
CBO-3	2.69	95.34	0.02305	0.965	89.56	0.04082	0.998
CBO-4	2.57	95.74	0.02272	0.942	85.24	0.03264	0.996
CBO-5	2.1	94.40	0.01662	0.874	86.08	0.03371	0.996
CBO-6	1.87	99.61	0.03324	0.929	93.32	0.04632	0.996

### 3.7. Electrocatalytic degradation stability

**Fig. 10:**
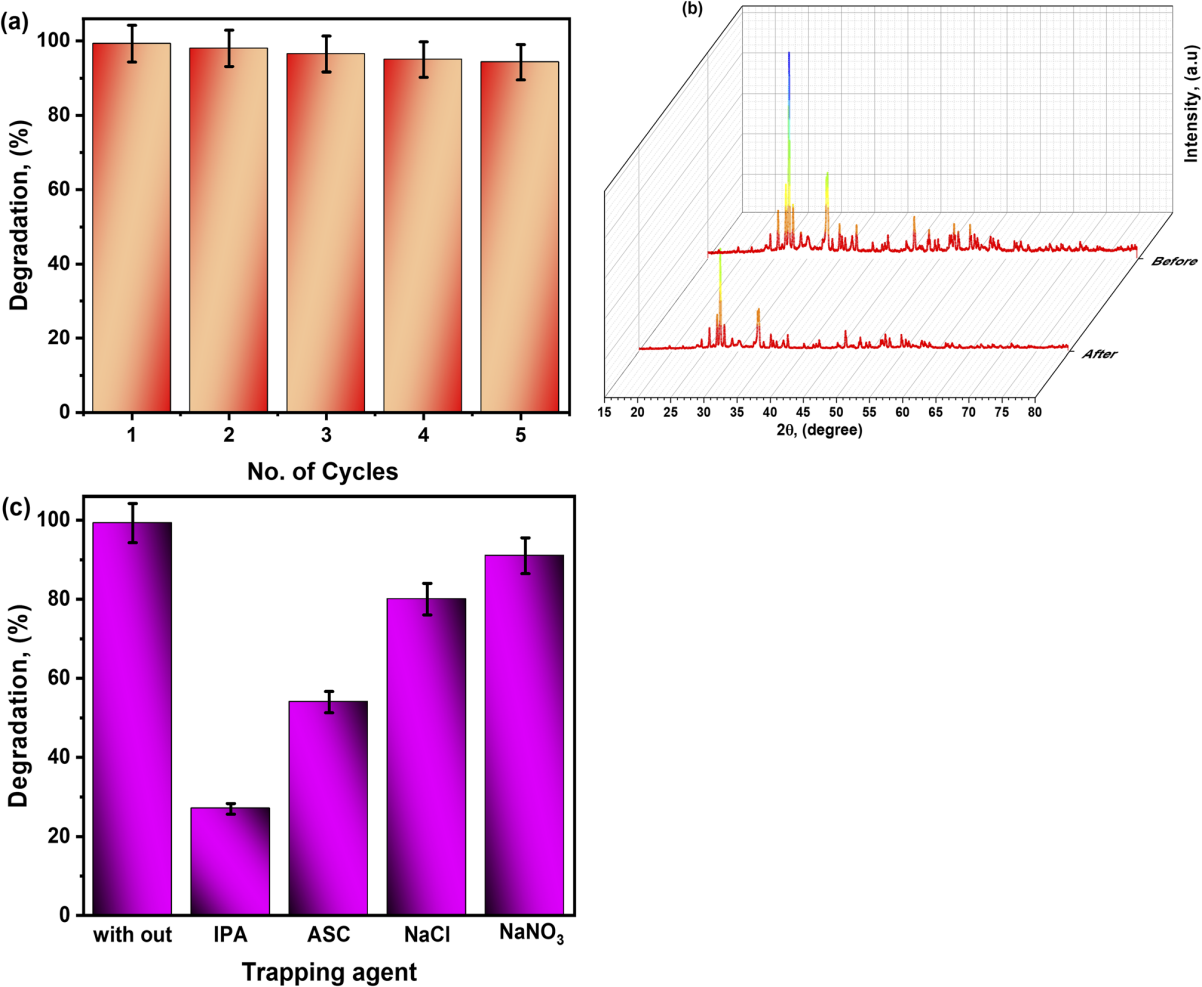
(a) Degradation efficiency of RhB with CBO-6 NCs after 5 cycles, (b) XRD of CBO-6 NCs after recycling, and (c) trapping agent of RhB dye with CBO-6 NCs.

The stability of electrocatalysts is crucial in the application sector. Five electrocatalytic cycles were conducted to examine the stability of the CBO-6 NC catalysts. After each cycle, the electrocatalyst was recovered by centrifugation, cleaned with distilled water and ethanol, and then dried in an oven heated at 80 °C. The electrocatalytic degradation stability of the as-prepared CBO-6 NCs was assessed by a cyclic degradation experiment (Fig. 10(a)). With the same starting concentration of pollutants in the degradation reaction solution, the catalyst utilized in the electrocatalytic degradation experiment goes through five successive cycles. The degradation activity of the CBO-6 NCs was shown to be stable even after five cycles, suggesting that the catalyst may be separated and used again. The degradation efficacy of RhB dye was found to have decreased from 99.28% to 94.5% following cycle 5. Catalysts are usually lost in trace amounts after recovery. These findings demonstrate that the NCs are an appropriate material for electrocatalytic breakdown of RhB, which mineralizes into carbon dioxide and water.^47^ The XRD results are shown in Fig. 10(b), which shows that during the five consecutive electrocatalytic cycle, the CBO-6 NC electrocatalysts retained their essential XRD diffraction peaks both before and after the electrocatalytic degradation process. This implies that the intensity of the diffraction peaks for the CBO-6 NCs decreases after the reusability test but their position does not change. The CBO-6 NCs can therefore be used as an efficient electrocatalyst for the treatment of wastewater.

### 3.8. Effects of radical scavengers

The findings of a trapping experiment to determine the main reactive species produced during the electrocatalytic breakdown of organic molecules are displayed in Fig. 10(c). Reactive species, *e.g.* O_2_˙^−^, OḢ, h^+^, and e^−^, were created by oxidation and reduction reactions on the valence and conduction band edges of electrocatalytic nanostructures through electrocatalytic processes. Accordingly, the reactive species produce degraded organic molecules in sequence. Scavengers, including sodium nitrate (e^−^), EDTA (h^+^), ascorbic acid (ASC, O_2_˙^−^), and isopropanol (IPA, OḢ), were previously studied to explore the validity of radical scavengers in the electrocatalytic degradation of RhB. The current findings agree with previous reports that detected OḢ as a key species in CBO-6 NCs *via* electrocatalytic degradation. These scavenger results display that ROS, *e.g.* hydroxyl radicals, play a major role in degrading organic contaminants into aqueous solutions.

### 3.9. Electrocatalytic reaction mechanism

**Fig. 11:**
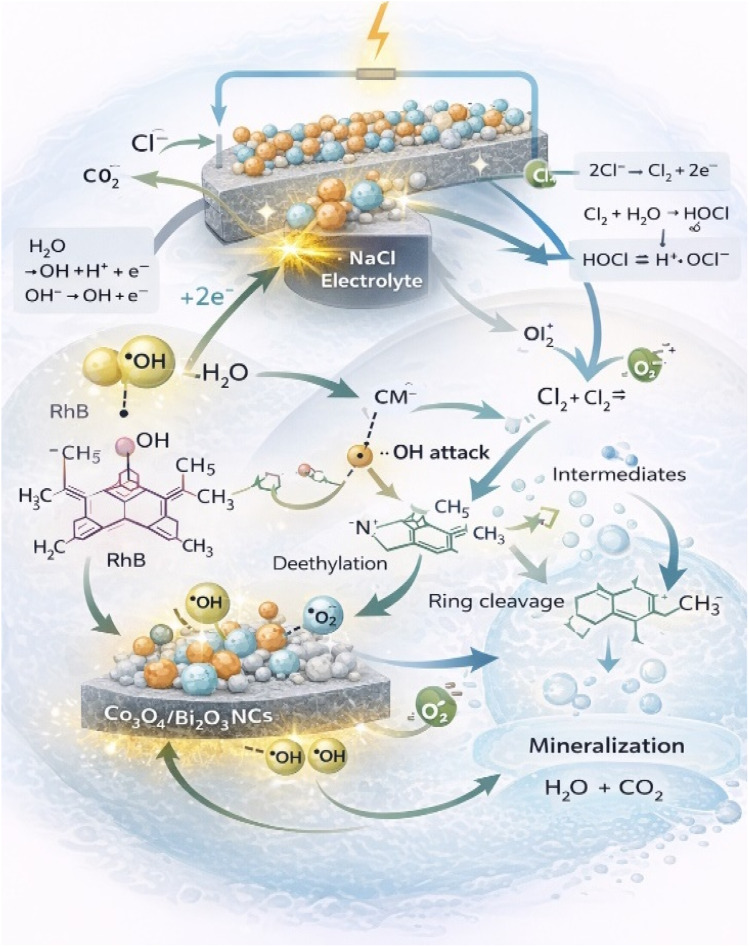
Mechanism diagram of the electrocatalytic degradation of RhB dye with CBO NCs.

Numerous ions, *e.g*. H^+^, Na^+^, OH^−^, Cl^−^, NO_3_^−^, SO_4_^2−^, and SO_3_^2−^, are frequently present in actual dye wastewater. This study examined the impact of NaCl, a common favorable electrolyte, on RhB degradation. The decolorization rate of RhB was clearly improved, whilst NaCl functioned as an electrolyte, since the produced Cl_2_ was transformed to ClO^−^ with intense oxidation (Fig. 11). The particular procedure was as follows:^48^

11 2Cl^−^ → Cl_2(aq)_ + 2e^−^

12 Cl_2(aq)_ + H_2_O → HClO^−^ + Cl^−^ + H^+^

13 HClO^−^ ↔ ClO^−^ + H^+^

14 CBO-6 NCs + hυ → h^+^ + e^−^

15 h^+^ + H_2_O → OḢ

16 RhB dye + OCl^−^ + OḢ + O_2_˙^−^ → CO_2_ + H_2_O + Cl^−^

## 4 Conclusions

In summary, Bi_2_O_3_ NPs and Co_3_O_4_-Bi_2_O_3_ nanocomposites were made by the sol–gel/auto-combustion method using diverse concentrations of Co_3_O_4_. The XRD results verified the monoclinic phase of Bi_2_O_3_. The addition of Co_3_O_4_ to Bi_2_O_3_ decreased the crystallite size, indicating significant structural changes in Bi_2_O_3_ upon Co_3_O_4_ incorporation. Morphological characterizations indicate that Co_3_O_4_-Bi_2_O_3_ consists mostly of mixed-morphology nanorods. The visible-light absorption capability of the Co_3_O_4_-Bi_2_O_3_ NCs was confirmed by UV-DRS analysis. The electrocatalytic degradation experiments clearly showed that 99.61% of RhB and 93.32% of DR 31 were degraded using CBO-6 NCs as the electrocatalyst. The scavenger investigation exhibits that the creation of ROS, *e.g.* hydroxyl radicals, plays a major role in degrading RhB dye. These findings confirm that Co_3_O_4_-Bi_2_O_3_ NCs are promising candidates for environmental remediation applications, mainly for effective electrocatalytic degradation of hazardous organic pollutants in wastewater.

## Author contributions

Nourhan A. M. Ragab: methodology, investigation, formal analysis, data curation. Faheem Shah, Esam Bakir and MY Nassar: methodology, investigation, formal analysis. Heba Y. Zahran: writing – original draft, visualization, validation. Elbadawy A. Kamoun: writing – review & editing, writing – original draft, visualization, validation, supervision, project administration. Heba Zahran, and V. Ganesh: software, resources, methodology, investigation. Ibrahim S. Yahia: writing – review & editing, writing – original draft, supervision, software, resources, project administration.

## Conflicts of interest

The authors declare that they have no known competing financial interests or personal relationships that could have appeared to influence the work reported in this paper.

## Supplementary Material

RA-OLF-D6RA04249J-s001

## Data Availability

Data will be made available on request. Supplementary information (SI) is available. See DOI: https://doi.org/10.1039/d6ra04249j.
