# Supplementary material for: Fabrication of heterojunctioned Co3O4-Bi2O3 nanocomposites and their sustainable electrocatalytic degradation of rhodamine B, and direct red 31 dyes
Source: RSC Adv. 2026 Jul 29;16(39):43744–58. doi: 10.1039/d6ra04249j (PMC13417793; doi:10.1039/d6ra04249j)
Supplement: Supplementary file 1 [file RA-016-D6RA04249J-s001.pdf]

Supplementary Information (SI) for RSC Advances.
This journal is © The Royal Society of Chemistry 2026

Supplementary Materials

Fabrication of heterojunctioned Co_3O_4-Bi_2O_3 nanocomposites and its sustainable electrocatalytic degradation of Rhodamine B, and Direct Red 31 dyes

Elbadawy A. Kamoun[1*], Nourhan A.M. Ragab[2], Heba Y. Zahran[3], Faheem Shah[1], M.Y. Nassar[1], Esam Bakir[1], V. Ganesh[3], Ibrahim S. Yahia[3*]

[1]*Department of Chemistry, College of Science, King Faisal University, Al-Ahsa 31982, Saudi Arabia.*
[2]*Nanotechnology and Catalysis Sections, Egyptian Company for Carbon Materials, El-Sheraton/El-Nozha, Cairo 11757, Egypt.*
[3]*Laboratory of Nano-Smart Materials for Science and Technology (LNSMST), Department of Physics, Faculty of Science, King Khalid University, P.O. Box 9004, Abha, Saudi Arabia.*
**Corresponding authors: ekamoun@kfu.edu.sa, badawykamoun@yahoo.com (E.A. Kamoun) Tel: +201283320302, and dr_isyahia@yahoo.com (I.S. Yahia).*

Table **S1**: Featured FTIR peaks of Co_3O_4-Bi_2O_3 NCs.

ν, (cm^{-1})	Vibrational mode
1386	C–O
844 - 564	Bi–O
561 - 665	Co-O

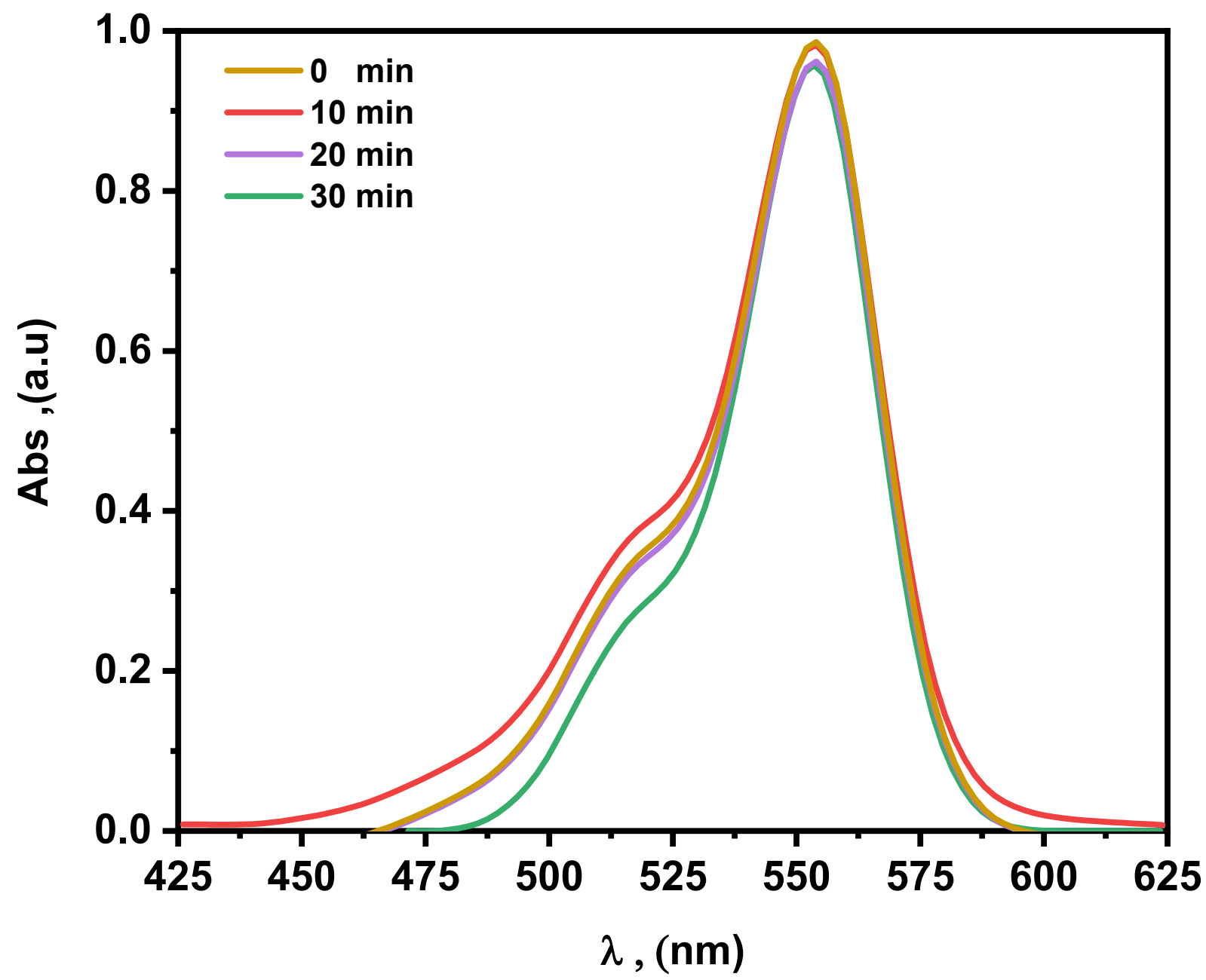

Fig. S1 Absorbance of RhB dye with CBO-6 NCs at adsorption process.
