# Supplementary material for: Fabrication of heterojunctioned Co3O4-Bi2O3 nanocomposites and their sustainable electrocatalytic degradation of rhodamine B, and direct red 31 dyes
Source: RSC Adv. 2026 Jul 29. Online ahead of print. doi: 10.1039/d6ra04249j (PMC13417793; doi:10.1039/d6ra04249j)
Supplement: RA-OLF-D6RA04249J-s001 [file RA-OLF-D6RA04249J-s001.pdf]

Supplementary Information (SI) for RSC Advances.
This journal is © The Royal Society of Chemistry 2026

Supplementary Materials

# Fabrication of heterojunctioned $Co_3O_4$-$Bi_2O_3$ nanocomposites and its sustainable electrocatalytic degradation of Rhodamine B, and Direct Red 31 dyes

Elbadawy A. Kamoun[1*], Nourhan A.M. Ragab[2], Heba Y. Zahran[3], Faheem Shah[1], M.Y. Nassar[1], Esam Bakir[1], V. Ganesh[3], Ibrahim S. Yahia[3*]

*[1]Department of Chemistry, College of Science, King Faisal University, Al-Ahsa 31982, Saudi Arabia.*
*[2]Nanotechnology and Catalysis Sections, Egyptian Company for Carbon Materials, El-Sheraton/El-Nozha, Cairo 11757, Egypt.*
*[3]Laboratory of Nano-Smart Materials for Science and Technology (LNSMST), Department of Physics, Faculty of Science, King Khalid University, P.O. Box 9004, Abha, Saudi Arabia.*
**Corresponding authors: ekamoun@kfu.edu.sa, badawykamoun@yahoo.com (E.A. Kamoun) Tel: +201283320302, and dr_isyahia@yahoo.com (I.S. Yahia).*

Table **S1**: Featured FTIR peaks of $Co_3O_4$-$Bi_2O_3$ NCs.

| ν, ($cm^{-1}$) | Vibrational mode |
|---|---|
| 1386 | C–O |
| 844 - 564 | Bi–O |
| 561 - 665 | Co-O |

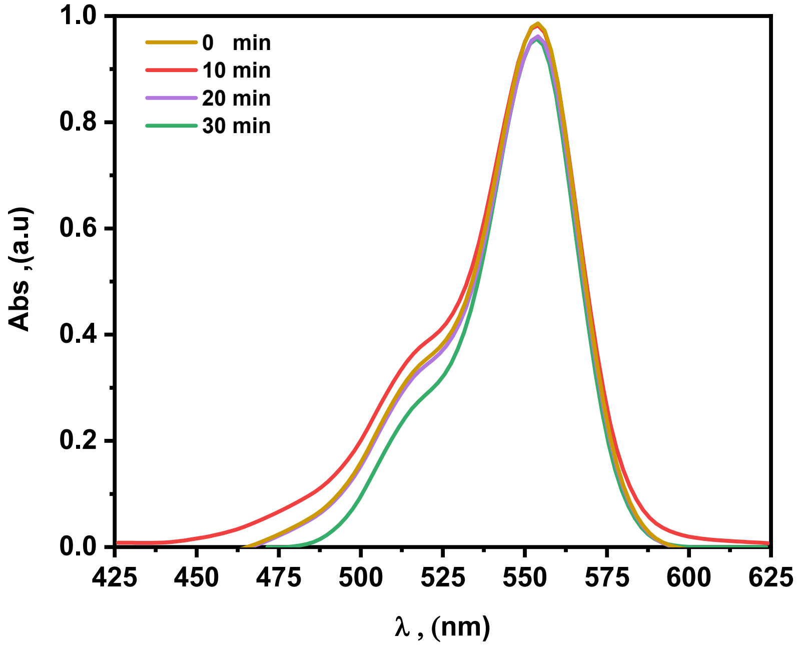


Fig. S1 Absorbance of RhB dye with CBO-6 NCs at adsorption process.
